# Augmentation of working memory training by transcranial direct current stimulation (tDCS)

**DOI:** 10.1038/s41598-017-01055-1

**Published:** 2017-04-21

**Authors:** Steffen Philipp Ruf, Andreas J. Fallgatter, Christian Plewnia

**Affiliations:** Department of Psychiatry and Psychotherapy, Neurophysiology & Interventional Neuropsychiatry, University of Tübingen, Calwerstrasse 14, 72076 Tübingen, Germany

## Abstract

Transcranial direct current stimulation (tDCS) to the dorsolateral prefrontal cortex (dlPFC) can modulate working memory (WM) performance. However, evidence regarding the enhancement of WM training, its sustainability and transferability is ambiguous. Since WM functioning appears to be lateralized in respect to stimulus characteristics, this study examined the difference between task-congruent (spatial-right, verbal-left), task-incongruent (spatial-left, verbal-right) and sham tDCS in regards to the efficacy of WM training. In a randomized, sham-controlled experiment, 71 healthy adults trained on a spatial or verbal adaptive n-back task. After a baseline session, anodal or sham tDCS (1 mA) to the right or left dlPFC was applied during the next three training sessions. Sustainability of training gains and near-transfer (verbal or spatial 3-back task) were tested in a fourth training and a follow-up session. Compared to sham stimulation, we found a steeper learning curve when WM training was combined with task-congruent tDCS. This advantage was also present compared to task-incongruent tDCS. Moreover, these effects lasted for up to nine months and transferred to the respective untrained task. These long-lasting, transferable, task-specific effects demonstrate a behaviorally relevant and sustainable facilitation of neuroplastic processes by tDCS that could be harnessed for the treatment of disorders associated with deficient WM.

## Introduction

Working memory (WM) functions are of decisive importance for effective human behavior. Deficits in WM play a crucial role in psychiatric disorders particularly schizophrenia and dementia^1, 2^. It has also been demonstrated that WM training, e.g. by repetition of a n-back task, can yield performance gains that transfer to untrained tasks^3, 4^. Moreover, WM training has been shown to have beneficial effects on cognitive control functions indicating potential applications in clinical, preventive, and occupational settings^5^. However, the amount of trainability of WM as well as the sustainability and generalization of training gains remains contentious^6, 7^. Therefore, approaches to improve the effectivity of WM training seem highly desirable. As a new technique to enhance cognitive capacities, transcranial direct current stimulation (tDCS) has gained increasing popularity and research interest in the past few years^8^. Based on comprehensive evidence for a polarity-dependent malleability of neuronal excitability^9, 10^, it has been shown that tDCS can modulate executive and cognitive functioning in healthy subjects e.g. ref. 11 as well as in patients with neurological e.g. ref. 12 and psychiatric disorders e.g. ref. 13. Regarding the enhancement of specific WM functions, anodal tDCS to the left dorsolateral prefrontal cortex (dlPFC) has been demonstrated to yield particularly beneficial effects^14^ by means of concurrent ‘online’ or preceding ‘offline’ stimulation^15–17^. Nevertheless, recent meta-analyses question the robustness and validity of tDCS-effects on cognitive functioning in general, including WM^18–20^. Moreover, when evaluating the effect of tDCS on WM, it is important to discriminate between a transient enhancement of performance and a facilitation of learning processes. First evidence for the latter was provided by coupling tDCS with implicit motor learning^21^. These findings have been extended by showing that tDCS and transcranial random noise stimulation (tRNS), linked with multi-session learning, can actually induce long-term enhancement of memory functions^22^ and associated brain activity^23^. In parallel, it has also been demonstrated in animal models that repeated sessions of tDCS can support WM learning and facilitate long-term neuroplastic processes^24^. Until recently, studies analyzing tDCS effects on training gains across multiple sessions were scarce and, although indicating small effects on WM function, did not consistently support the notion of facilitative tDCS-effects on the practice-dependent improvement of WM as, for instance, represented by differences in learning curves^25–27^. However, current studies provide new evidence that anodal stimulation of the dlPFC actually results in enhanced learning during a WM training^28^ and that these effects can transfer to untrained tasks^29^. Nevertheless, a high variability of stimulation parameters and study settings as well as the complex interaction between task specific neuronal activity and stimulation impede the comparability of the results^30, 31^. Based on this notion, the consideration of asymmetries in the lateral organization of WM^32, 33^ might improve the efficacy of tDCS on WM^34^. Imaging and stimulation studies actually suggest a preferential left- and right-sided activation during verbal and spatial WM demands, respectively^35–39^. Moreover, inter-individual differences in task-proficiency and associated cortical activity may critically influence the effect of tDCS^40^.

Therefore, in this study, we tested the hypotheses that anodal tDCS (i) enhances learning and sustainability of the training gains, (ii) facilitates transfer effects to an untrained task and (iii) that these effects particularly emerge when the stimulation is targeted congruent with the domain-specific organization of WM, i.e. tDCS to the right dlPFC during spatial and tDCS to the left dlPFC during verbal WM training.

## Results

### Effects on the training task during training

**Figure 1:**
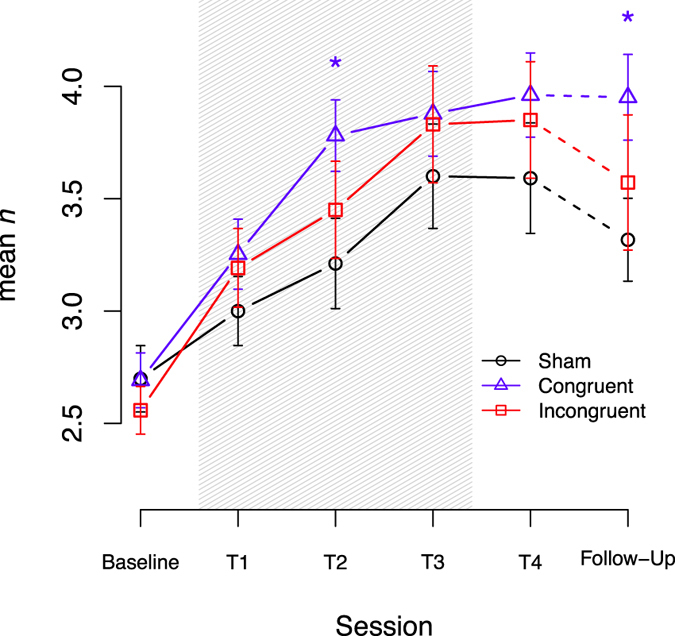
Adaptive n-back training (mean n) across sessions with T1 – T3 combined with anodal tDCS during one week. At T4, the adaptive n-back was trained without tDCS. Follow-up sessions were performed without tDCS. Shading indicates application of tDCS. Error-bars represent the standard error (SEM). *Indicates significant t-tests (p < 0.05).

**Figure 2:**
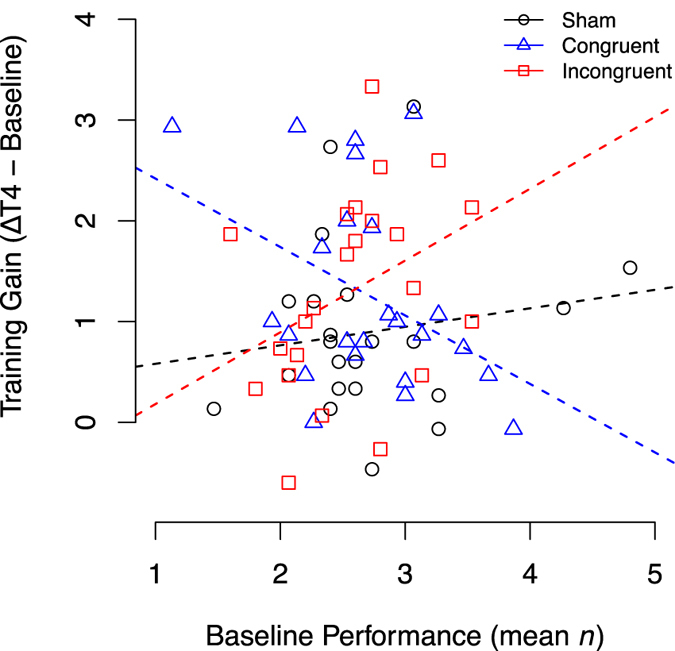
Training gains (performance at T4 minus baseline performance) in dependency of baseline performance. Dashed lines are linear regression lines for each group.

Regression parameters of the linear mixed-effect model indicate that performance and the benefits of WM training are baseline dependent (β_baseline_ = 0.64, SE = 0.18, z = 3.52, p < 0.001; β_session×baseline_ = 0.17, SE = 0.06, z = 2.67, p = 0.007). In subjects that received *task-congruent tDCS* (right dlPFC during spatial or left dlPFC during verbal WM training), the interaction between session and stimulation revealed increased learning rates compared to the sham-group (β_session×CONGRUENTvsSHAM_ = 1.00, SE = 0.27, z = 3.66, p < 0.001). However, no significant difference was observed in the subjects that received *task-incongruent* tDCS (right dlPFC during verbal or left dlPFC during spatial WM training; β_session×INCONGRUENTvsSHAM_ = −0.02, SE = 0.29, z = −0.08, p = 0.936). Additionally, the increased learning rate of the congruent-group is dependent on baseline performance, as seen in the significant threefold interaction for the congruent-group (β_session×CONGRUENTvsSHAM×baseline_ = −0.37, SE = 0.10, z = −3.74, p < 0.001). For the *task-incongruent tDCS*, no such interaction was found (β_session×INCONGRUENTvsSHAM×baseline_ = 0.03, SE = 0.11, z = 0.24, p = 0.810). Therefore, participants with lower baseline performance receiving *task-congruent tDCS* profited more from stimulation. Comparing the effects of *task-congruent* with *task-incongruent tDCS*, the advantage of *task-congruent tDCS* was maintained (β_session×CONGRUENTvsINCONGRUENT_ = 1.02, SE = 0.30, z = 3.36, p < 0.001; β_session×CONGRUENTvsINCONGRUENT×baseline_ = −0.39, SE = 0.11, z = −3.47, p < 0.001). All other regression parameters didn’t reach statistical significance. Learning curves, i.e. progression across all sessions (including follow-up), are depicted in Fig. 1. Post-hoc t-tests revealed that, compared to sham tDCS, subjects with *task-congruent tDCS* achieved superior training gains at session T2 (t(45) = 3.29, p = 0.002). Comparisons between groups across all timepoints are given in Table 1. Interaction of baseline performance and training gains, i.e. learning progress across sessions is illustrated in Fig. 2.

**Table 1:** Pairwise t-Test comparisons between groups across all training sessions.

Time	Group			Δ Congruent vs. Sham			Δ Incongruent vs. Sham			Δ Congruent vs. Incongruent		
	Sham	Congruent	Incongruent	t-Value (df)	p-Value	Cohen’s d	t-Value (df)	p-Value	Cohen’s d	t-Value (df)	p-Value	Cohen’s d
Session 1	3.00 (0.74)	3.25 (0.76)	3.19 (0.86)	1.50 (45)	0.141	0.44	1.92 (45)	0.061	0.56	−0.42 (46)	0.674	0.12
Session 2	3.21 (0.96)	3.78 (0.78)	3.45 (1.06)	3.29 (45)	**0.002**	0.96	1.78 (45)	0.082	0.52	0.92 (46)	0.361	0.27
Session 3	3.60 (1.11)	3.88 (0.92)	3.83 (1.27)	1.18 (45)	0.246	0.34	1.54 (45)	0.132	0.45	−0.31 (46)	0.761	0.09
Session 4	3.59 (1.18)	3.96 (0.92)	3.85 (1.27)	1.41 (45)	0.166	0.41	1.50 (45)	0.141	0.44	−0.08 (46)	0.938	0.02
Follow-Up	3.32 (0.83)	3.95 (0.74)	3.57 (1.31)	2.96 (33)	**0.006**	1.01	1.72 (37)	0.093	0.55	0.86 (32)	0.394	0.30

Data are mean values with standard deviations in parentheses; Comparisons are made with baseline corrected mean values (Δ) at respective time points.

### Effects on the training task at follow-up

The ANCOVA for the adaptive n-back of the follow-up session again revealed a significant effect of baseline (F(1, 48) = 15.14, p < 0.001, η^2^ = 0.14, η_p_^2^ = 0.24), group F(2, 48) = 6.91, p < 0.002, η^2^ = 0.13, η_p_^2^ = 0.22) and a baseline × group interaction F(2, 48) = 6.72, p < 0.003, η^2^ = 0.12, η_p_^2^ = 0.22). Overall, baseline performance predicts follow-up performance (β_baseline_ = 0.84, SE = 0.22, t(48) = 3.89, p < 0.001). At the follow-up session participants who received *task-congruent tDCS* showed greater performance gains as compared to the sham group (β_CONGRUENTvsSHAM_ = 2.02, SE = 1.01, t(48) = 2.01, p = 0.050), regardless of baseline performance (β_baseline×CONGRUENTvsSHAM_ = −0.51, SE = 0.37, t(48) = −1.38, p = 0.17). Additionally, the group with *task-congruent tDCS* showed greater performance gains when compared to the *task-incongruent* group in a baseline-dependent manner (β_CONGRUENTvsINCONGRUENT_ = 4.12, SE = 1.11, t(48) = 3.71, p < 0.001; β_baseline×CONGRUENTvsINCONGRUENT_ = −1.50, SE = 0.42, t(48) = −3.57, p < 0.001). However, comparing the effects of *task-incongruent tDCS* to the *sham tDCS* (β_INCONGRUENTvsSHAM_ = −2.10, SE = 0.97, t(48) = −2.17, p = 0.035; β_baseline×INCONGRUENTvsSHAM_ = 0.99, SE = 0.37, t(48) = 2.71, p = 0.009) indicates a baseline dependent advantage of the *task-incongruent tDCS* over sham. Simple t-tests of performance differences at follow-up reveal a superiority of the congruent-group compared to sham (t(33) = 2.96, p = 0.006).

### Effects on the transfer task

**Figure 3:**
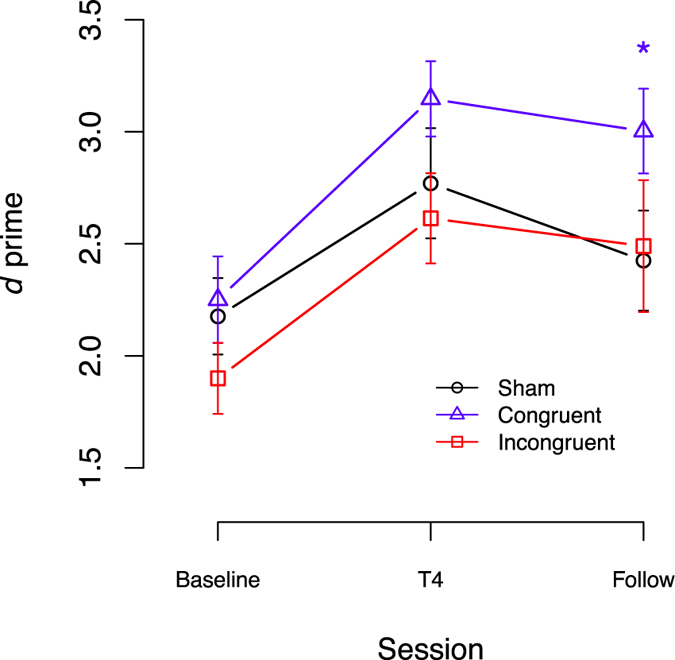
At baseline, T4, and T5, performance (d′) on the 3-back near-transfer task was measured without concurrent tDCS to test the transfer of tDCS-enhanced adaptive n-back training gains. Error-bars represent the standard error (SEM). *Indicates significant t-tests (p < 0.05).

The ANCOVA for the 3-back regarding performance at session T4 showed a significant effect of baseline (F(1, 65) = 37.23, p < 0.001, η^2^ = 0.27, η_p_^2^ = 0.36), group (F(2, 65) = 6.14, p = 0.003, η^2^ = 0.09, η_p_^2^ = 0.16) and the baseline × group interaction (F(2, 65) = 5.11, p = 0.010, η^2^ = 0.07, η_p_^2^ = 0.13) (Fig. 3). Participants with greater baseline performance reached higher post session scores (β_baseline_ = 1.15, SE = 0.19, t(65) = 6.10, p < 0.001). Only participants of the *task-congruent* group showed a greater improvement of performance compared to the sham-group (β_CONGRUENTvsSHAM_ = 1.96, SE = 0.58, t(65) = 3.35, p = 0.001). Investigation of the interaction revealed that participants with lower pre-session performance benefited more from the stimulation (β_baseline×CONGRUENTvsSHAM_ = −0.74, SE = 0.25, t(65) = −3.00, p = 0.004). Additionally, when contrasting *task-congruent* to the *task-incongruent* tDCS, the differences remained significant (β_CONGRUENTvsINCONGRUENT_ = 1.36, SE = 0.55, t(65) = 2.45, p = 0.017; β_baseline×CONGRUENTvsINCONGRUENT_ = −0.51, SE = 0.25, t(65) = −2.04, p = 0.046). Comparing the *task-incongruent* tDCS to sham, no significant differences could be identified (β_INCONGRUENTvsSHAM_ = 0.60, SE = 0.59, t(65) = 1.01, p = 0.314; β_baseline×INCONGRUENTvsSHAM_ = −0.23, SE = 0.27, t(65) = −0.85, p = 0.398).

At the follow-up session the ANCOVA for the 3-back showed a significant effect of baseline (F(1, 48) = 9.04, p = 0.004, η^2^ = 0.09, η_p_^2^ = 0.16). The effects of group (F(2, 48) = 1.17, p = 0.32, η^2^ = 0.02, η_p_^2^ = 0.05) and the baseline × group interaction (F(2, 48) = 2.05, p = 0.14, η^2^ = 0.04, η_p_^2^ = 0.08) didn’t reach statistical significance (Fig. 3). Regression parameter revealed that participants with greater baseline performance reached higher post session scores (β_baseline_ = 0.61, SE = 0.20, t(48) = 3.01, p = 0.004). However, simple t-tests for differences in performance of the untrained near-transfer task revealed an advantage of the congruent-group when compared to sham (t(33) = 2.35, p = 0.025).

### Correlation between baseline performance and sample characteristics

The linear regression revealed a negative relationship between age and baseline performance of the adaptive n-back task (β_age_ = −0.07, SE = 0.03, t(65) = −2.56, p = 0.013). All other predictors (education, sex, right-handedness and IQ) did not reach statistical significance (p > 0.05).

### Proof of blinding

For comparing group-allocation guesses of the participants, a χ^2^-test was applied. It revealed no differences between guesses of the three groups (χ^2^(2) = 0.76, p = 0.684), which indicates successful blinding of stimulation.

### Analysis of mood changes

Data of one participant had to be removed for the analysis of mood changes due to missing values. Examining the ANOVA of the positive scale, neither group (F(2, 80) = 0.31, p = 0.738), nor session (F(2, 82) = 1.02, p = 0.364), nor the group × session interaction (F(4, 80) = 0.94, p = 0.447) showed significance. Likewise, the ANOVA for the negative scale revealed no significant differences (group: F(2, 80) = 0.51, p = 0.600; session: F(2, 82) = 1.09, p = 0.341; group × session: F(4, 80) = 0.30, p = 0.880).

### Reported adverse effects

Adverse effects reported by the participants are displayed in Table 2. For each adverse effect, a Kruskal-Wallis test was calculated to check for group differences. Analysis revealed no significant differences between groups regardless of type of adverse effect, indicating no noxious effects of stimulation and a successful sham arrangement.

**Table 2:** Adverse effects of tDCS.

Adverse effect	Sham	Congruent	Incongruent	Kruskal-Wallis	p-value
Tingling (electrode)	2.91 (1.12)	2.96 (0.95)	2.79 (0.98)	X^2^(2) = 0.40	0.817
Tingling (head area)	1.35 (0.83)	1.25 (0.68)	1.17 (0.48)	X^2^(2) = 0.33	0.847
Fatigue	1.78 (1.13)	1.71 (1.00)	1.67 (1.01)	X^2^(2) = 0.04	0.980
Itching	2.13 (1.25)	2.25 (1.26)	2.04 (1.12)	X^2^(2) = 0.29	0.863
Headache	1.35 (0.65)	1.46 (0.83)	1.33 (0.70)	X^2^(2) = 0.21	0.900
Nausea	1.17 (0.58)	1.04 (0.20)	1.04 (0.20)	X^2^(2) = 0.69	0.709

Data are mean values of a 1–5 Likert scale with standard deviations in parentheses.

## Discussion

The present study examined the effects of anodal tDCS to the left and right dlPFC during spatial and verbal WM training on the learning effect across practice sessions and the transfer of improvements to untrained WM tasks. The key results are first that enhancement of dlPFC activity by anodal tDCS can improve WM learning curves. Second that the beneficial effects of tDCS-enhanced training can transfer to an untrained domain. Third that the tDCS-dependent learning gains are sustained for up to nine months. Fourth, that lower pre-training performance predicts higher benefits from stimulation. And fifth, that the effects on WM training particularly emerge when the stimulus specific laterality of WM functioning is accounted for.

At large, these data provide new evidence in support of the notion that tDCS can enhance the efficacy of WM training^18, 20^. Nevertheless, the necessary pre-specification of stimulation and training parameters (e.g. intensity, location and polarity of stimulation, number of training sessions and inter-training intervals) limit the generalization of the results^30, 31^. It is quite unlikely that the parameters used here are the best possible but optimization of the stimulation parameters was beyond the scope of this study. However, the task- and laterality-specific effects that were predicted by the notion of a hemispheric organization of WM^39^ support the reliability of the findings. This specification of the stimulation targets was based on a multitude of functional neuroimaging studies that have identified the dlPFC as a central hub of the neuronal network underlying WM functions^41^. Additionally, a range of other cortical areas are involved, including bilateral superior and inferior frontal gyri, (pre-) supplementary motor area as well as parietal areas^42^. However, non-invasive brain stimulation studies using repetitive transcranial magnetic stimulation (rTMS) and tDCS have focused on the dlPFC to support healthy and disturbed WM^18^. As tDCS appeared to be beneficial for WM performance, the question arises if the learning progress as such can be enhanced by tDCS. In this regard, several studies indicated that the performance during WM trainings can be facilitated by tDCS^26, 27, 29, 43–45^. But unambiguous evidence for improved learning progress across multiple sessions and sustainability of training gains is still scarce^28^. In this respect, showing differences in learning curves between tDCS and sham stimulation parallel to WM practice, our findings provide relevant additional proof for a sustainable improvement of WM training effects by tDCS. However, it has to be considered that the follow-up measures were obtained 3 and 9 months after the training of spatial and verbal WM, respectively. Therefore, conclusions on the temporal stability of training effects are preliminary.

Regarding the transfer of stimulation-enhanced training gains to untrained tasks, available studies provide inconsistent results. Transfer effects were reported comparing a tDCS-only (without training) group with a tDCS and training group^43^ or comparing an active tDCS group with a no-contact control group^26^. Recently, transfer effects to another spatial task^28^ and, in older subjects, far transfer gains after one month^29, 45^ were documented after tDCS to the right dlPFC parallel to spatial WM training.

Moreover, the optimal stimulation timing is largely unclear. Recent meta-analyses on differential effects of online and offline tDCS on WM performance come to conflicting results^18, 20, 30^. However, based on our own experience^46–48^ and other previous studies^25^ we used tDCS parallel to WM training and thus confirm the efficacy of the direct combination of tDCS and cognitive training. In order to improve the precision of our intervention, we followed up on considerations attributing the lack of tDCS-effects on spatial WM after stimulation to the left dlPFC^26^. Considering the domain-specific lateralization of WM functions with the left dlPFC particularly involved in verbal and the right dlPFC in spatial WM functioning^33, 35, 36^, we performed a crosswise comparison of task-congruent with task-incongruent and sham stimulation. The electrode placement over left (F3) or right (F4) dlPFC together with an extracephalic return electrode, enabled us to test the hypothesis that, for effective enhancement, anodal tDCS has to be targeted to domain-specific active dlPFC (left for verbal, right for spatial stimulus material). These results, in line with previous studies^49, 50^, support the notion of an at least partially left- and right-lateralization of verbal and spatial WM. Of course, this dichotomy of verbal and spatial WM pinpoints only a small aspect of the much more complex functional organization of the networks subserving WM processes^51^. For instance, the very recent study of Au *et al*.^28^ demonstrated training-enhancement by both tDCS of the left and right dlPFC. However, in this study only visuospatial WM was trained, comprising seven training sessions and applying 2 mA anodal tDCS. Therefore, it is quite possible that compared to our study, longer training and higher stimulation intensities might overcome the laterality effects. But, consistent with our findings, in the study of Au *et al*. transfer effects were only found after the task-congruent right sided stimulation. Therefore, regarding transfer effects, our results advocate the idea that tDCS should be targeted to the cortical area that is involved in the training task and not the task to which a transfer of performance is intended. This observation is again in line with the notion that tDCS should be linked with cortical activity^25^ to yield appropriate effects. Hence, translation of tDCS-enhanced WM trainings into clinical and therapeutic settings should consider this left-right lateralization of working memory functioning. Performance improvements and transfer effects induced by tDCS may only be achievable when the location of electrodes match the specific stimuli of the training task. However, further studies are needed to investigate under which circumstances the transfer of tDCS-induced learning effects appear robustly.

Furthermore, our results indicate that effects of tDCS are dependent on the pre-training performance. Participants with relatively low WM ability benefit the most, while participants with higher WM performance profit less from task-congruent stimulation. This contrasts with our observation that the subjects with higher WM proficiency at baseline generally show better training effects. Presumably, these individuals are more likely to activate the required neuronal resources. Accordingly, in these subjects, adding activation by tDCS would be less effective. In this regard, it must be noted that in our study, participants in the sham group were slightly older (mean age: 27 in the sham group vs. 23 and 24 years in the active groups). This might be relevant since, at baseline, age correlates negatively with performance. However, performance at baseline did not differ between groups. Moreover, differential effects were also found on training between congruent and incongruent stimulation as well as comparing performance between sham and task-congruent tDCS at follow-up, discounting baseline performance. Nevertheless, in general, the interaction between baseline performance and the efficacy of tDCS is complex. For example, it has been demonstrated that in an older group of subjects, participants with higher levels of education benefited more from tDCS during a WM task than adults with lower education^52^ indicating that strategy and motivation influence the effectiveness of tDCS^53^. Therefore, a direct link between our results – that lower baseline performance predicts better TDCS effects – and promising clinical use in subjects with lower baseline performance is premature and needs more specific empirical data.

Similarly, the idea that additional brain activation by anodal tDCS generally leads to enhanced behavioral performance has not always proved to be correct^54^. In healthy populations especially, a non-linear effect of stimulation intensity was reported^55^, i.e. a lower current intensity of 1 mA can, performance-wise, be superior to stimulation with 2 mA. These data, our own respective findings^46–48, 56, 57^, and the more reliable sham control, prompted us to use 1 mA stimulation intensity, despite previous and recent studies showing effects on WM with 2 mA stimulation^28, 29, 34, 58, 59^. However, patients with Parkinson’s disease^60^ and schizophrenia^61^ seem to benefit more from increased current intensity in regards to cognitive performance.

Furthermore, high inter-individual variability and the possibility of non-responders in regards to the efficacy of tDCS, e.g. caused by COMT gene or other polymorphisms, have to be considered when effects of tDCS vary^62–64^. Thus, future studies should place special emphasis on the elucidation of possible moderator variables and their impact in regards to tDCS efficacy.

In summary, the current study endorses the notion that tDCS can augment WM training if applied in a task- and laterality-dependent manner, specifically to the right dlPFC in spatial and the left dlPFC in verbal WM training. Moreover, these beneficial effects of tDCS can transfer to an untrained task and are observable for up to nine months. This proof of concept could help to develop effective tDCS-enhanced training regimens for the application in therapeutic settings.

## Materials and Methods

### Ethical Statement

The study was performed in accordance to the Declaration of Helsinki and approved by the ethics committee of the Medical Faculty of the Eberhard Karls University Tübingen. Prior to the conduction of the study, all participants gave their written informed consent. Participants received a small monetary compensation for their participation in the study.

### Subjects

Overall, 81 participants were recruited for both training schedules. They were randomly allocated to one of three groups by means of a computer-generated table: anodal stimulation to the left dlPFC, anodal stimulation to the right dlPFC, and sham stimulation. Nine participants dropped out before finishing all sessions. Data of one participant was excluded because n-back values were more than three interquartile-ranges above the upper quartile. In total, 71 participants (M = 24.45 years, SD = 5.16, 57 female, 14 male) completed all training and measurements sessions and were included in the statistical analysis. The first 36 individuals (M = 23.5 years, SD = 3.39, 30 female) were subjected to a spatial and the latter 35 (M = 25.4 years, SD = 6.40, 27 female) to a verbal WM training. Participants were checked for the following exclusion criteria: left-handedness (assessed by the Edinburgh Handedness Inventory^65^ with a cut-off score of 50), current psychopharmacological medication or psychotherapeutic treatment, mental or neurological disorders, brain implants, or history of seizures. All participants had corrected to normal or normal vision. A detailed description of the study sample is given in Table 3.

**Table 3:** Sample descriptive data and baseline comparisons between groups.

Measure	Sham	Congruent	Incongruent	ANOVA/χ^2^	p-value
Gender (female/male)	18/5	18/6	21/3	X^2^(2) = 1.27	0.529
Age	26.74 (7.00)	22.96 (2.03)	23.75 (4.67)	F(2,68) = 3.77	0.028
Education (in years)	18.12 (4.25)	16.08 (1.81)	16.33 (2.83)	F(2,68) = 2.98	0.058
MWT-B	103.52 (14.54)	101.33 (10.20)	99.79 (8.15)	F(2,68) = 0.65	0.524
EHI (laterality-quotient)	93.00 (13.85)	88.75 (13.85)	92.63 (12.86)	F(2,68) = 0.72	0.49
Training task (mean n)	2.70 (0.71)	2.69 (0.60)	2.56 (0.52)	F(2,68) = 0.40	0.674
Transfer task (d-prime)	2.18 (0.82	2.25 (0.95)	1.90 (0.78)	F(2,68) = 1.13	0.329

Data are mean values with standard deviations in parentheses; EHI = Edinburgh Handedness Inventory; MWT-B = Multiple Choice Word Test-B.

### Procedure

**Figure 4:**
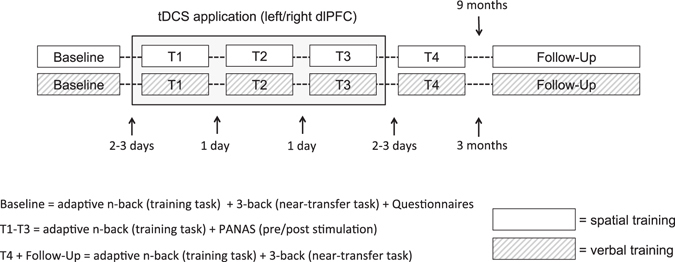
Procedure, task assessment, and timeline of the two training schedules.

The training schedules were performed in a sham-controlled, single-blinded training design with a *baseline* session, three tDCS-supported training sessions (*T1-T3*), a post-tDCS training session (*T4*) and a *follow-up* session (Fig. 4). The training started 2–3 days after baseline and was carried out during one week with 1 day (T1-T3) and 2–3 days (T4) inter-session intervals. The follow-up session took place nine and three months after spatial and verbal training, respectively. The baseline session was comprised of the questionnaires and the baseline assessment of the adaptive n-back and the near-transfer 3-back task. During T1-T3 participants trained the adaptive n-back task parallel to tDCS according to their group allocation (left dlPFC, right dlPFC, sham). The subjects were trained either on an adaptive spatial or verbal n-back task and performed a spatial or verbal 3-back as a near-transfer task, respectively. At T1-T3, before and after the training, the current affective state of the participants was assessed by the PANAS^66^ to evaluate possible stimulation dependent changes of affect. At T4 and follow-up, participants performed the adaptive n-back training without tDCS and 3-back near-transfer task. To ensure blinding of tDCS, the participants were asked to guess if they received active or sham stimulation. They also rated the adverse effects of stimulation on a 1 to 5 Likert-scale^67^ including questions on the most frequent adverse effects of tDCS: itching, tingling (near electrode and overall head area), headache, nausea, and fatigue.

### Transcranial Direct Current Stimulation

A CE-certified DC-Stimulator MC (NeuroConn GmbH, Ilmenau, Germany) was used to administer anodal direct current on the three training sessions. The current was applied via a pair of rubber electrodes (5 × 7 cm, 35 cm^2^) which where coated in adhesive conducting paste (10/20 conductive EEG paste, Kappamedical, USA). Electrode resistances were kept < 8 kΩ. In the active conditions, tDCS (1 mA) was administered for a total period of 20 min with the anode placed on F3 or F4 according to randomization (international 10–20 system^68^, longer side of the electrode positioned horizontally). The reference electrode was placed on the contralateral deltoid muscle (longer side of the electrode, positioned horizontally) to avoid unwanted stimulation effects on brain activity of other cortical regions^48, 69^. Modeling data indicates that the distribution of current density with this electrode placement is focused on the dlPFC^48^. In the sham group, stimulation was also applied to the left or right dlPFC (allocation 50/50) for 40 seconds to induce similar sensations on the scalp without relevant modulation of neural activity. In all three groups, stimulation was ramped up and down for 5 seconds.

### Training Tasks

**Figure 5:**
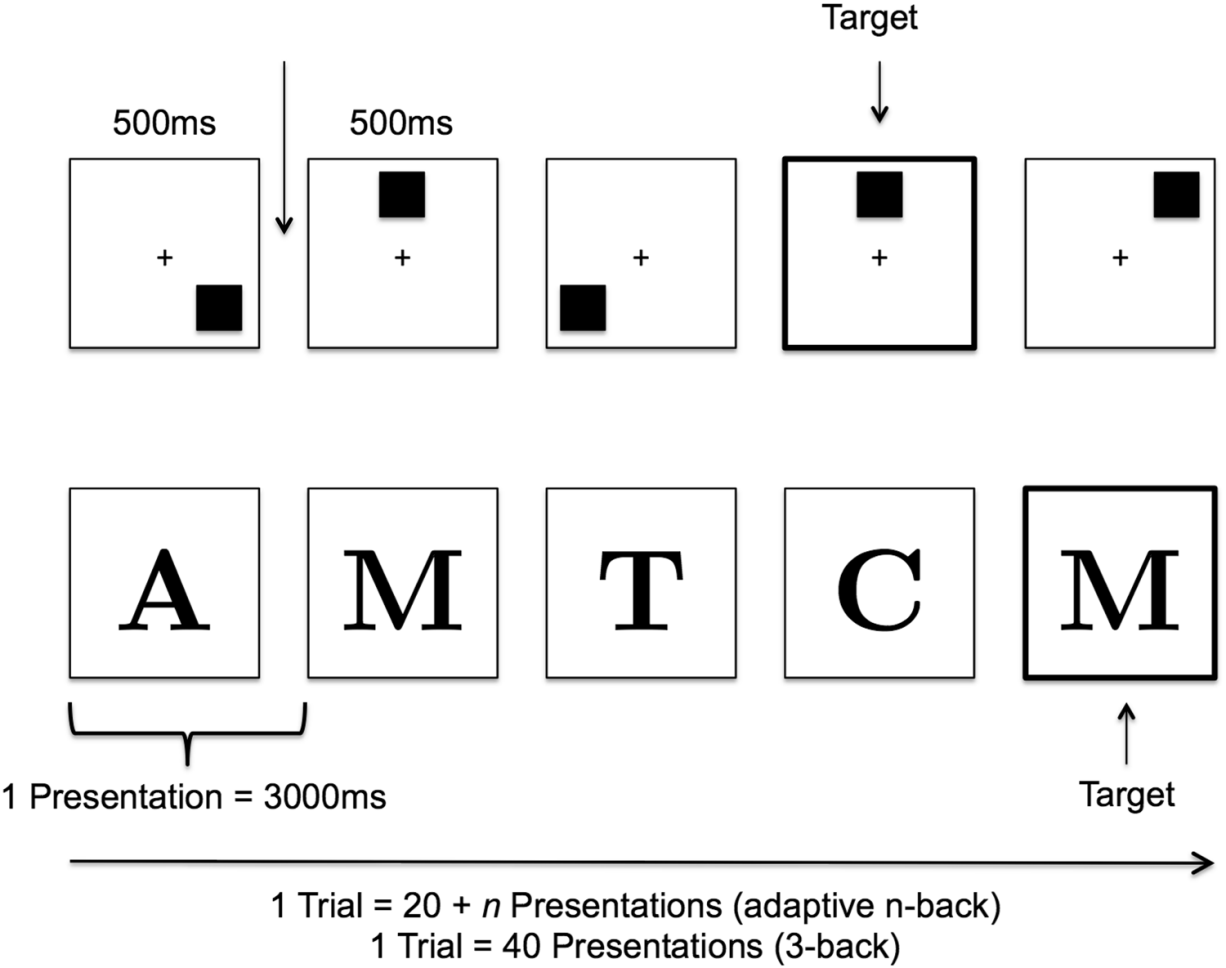
Schematic depiction of the training and transfer tasks (Figure adapted from Jaeggi *et al*., 2008).

For WM training, we used an adaptive n-back paradigm^3, 70–72^. Stimuli were presented using the open-source software Brain Workshop v4.8.4^73^ and PsychoPy2 v1.80.04^74^. In the adaptive spatial n-back, blue squares were presented subsequently on a black 3 × 3 grid (except the center position). Participants had to respond with a key press (space bar) if the current position of the blue square matched the position n-presentations before (target). In case of a mismatch the key press had to be omitted. The position of the squares varied randomly. In the adaptive verbal n-back, stimuli were sets of letters containing 8 letters randomly selected out of the complete alphabet (A-Z). The letters were randomly presented in a succeeding order and the participants had to press a key (space bar) when the current letter matched the letter n-presentations before (target). A schematic representation of the tasks is depicted in Fig. 5. Participants were instructed to react as accurately and as fast as possible. As stimulus selection occurred randomly, on average every eighth presentation was a target. The tasks consisted of 20 trials with each trial comprised of 20 + n presentations. As the task was adaptive, the task difficulty n varied per trial. Every participant started the task on every session with n = 1. After each trial they received feedback concerning their performance, with which the difficulty n for the upcoming trial was adapted: (1) with a score of <  = 50%, task difficulty n was decreased (n − 1); (2) in case of a score between 50% and 70%, n remained unchanged; (3) scoring > 70%, n was increased (n + 1). The score for each trial was calculated as follows: score = hit/(hit + miss + false alarm). The outcome measure for the training task was mean n, averaged over the last 15 out of 20 trials^75^.

### Near-transfer Task

To measure transfer to untrained tasks in a similar context (near transfer) and to test the transferability between WM domains, a verbal 3-back task was used if the adaptive training task was spatial and vice-versa^4, 14, 76^. The transfer tasks were programmed using PsychoPy2 v1.80.04^74^. Stimuli were the same as in the adaptive n-back tasks. The stimuli were randomly presented in a succeeding order and the participants had to press a key (space bar) when the current stimulus matched the stimulus three presentations before (target). In case of a mismatch the key press had to be omitted. The task itself consisted of five trials with 40 stimulus presentations per trial (Fig. 5) and a practice trial to get familiar with the task. In each trial, a different set of stimuli, i.e. positions of squares or letters, was used. Participants were instructed to react as accurately and as fast as possible. Due to its favorable psychometric properties^77^ d’-prime was defined as the outcome measure. It is calculated by subtraction of the standardized (z-transformed) hit-rate and false-alarms: d’ = z(HIT)−z(FA)^78, 79^.

### Statistical Analysis

Statistical analysis was conducted using the software R Version 3.3.1^80^ including the packages lme4^81^ and multcomp^82^. To examine the effects of tDCS on across-session learning (T1-T4) of the adaptive n-back tasks, linear mixed-effect models were fitted. For each model the dependent variable was the performance outcome (mean n) and the following fixed effects were entered: session (metric), group (categorical), baseline (metric) and all corresponding interactions. To account for unsystematic individual differences a random-intercept and a random-slope was entered. Linear mixed-effects models were chosen over an ANOVA-framework to allow for a detailed evaluation of learning slopes in consideration of baseline performance by means of regression-coefficients. According to our hypotheses, the factor group was effect-coded with the sham-group being the reference of comparison. Regression parameters of the linear mixed-effect models and their corresponding z-value and significance are reported. It should be noted that, for interpreting differences between groups across the complete training, all regression-coefficients have to be added up. Accordingly, coefficients can be counterintuitively negative when they are canceled out by other positive coefficients, e.g. higher interactions. If significant differences in learning rates were found between tDCS and sham, post-hoc comparisons of training gains (i.e. baseline corrected performance) at specific sessions were performed by independent t-Tests (two-tailed).

To test the superiority of stimulation linked with the assumed task-dependent neuronal activity, we first compared learning in subjects receiving *task-congruent tDCS* (right dlPFC during spatial and left dlPFC during verbal WM training) with learning during *task-incongruent tDCS* (right dlPFC during verbal and left dlPFC during spatial WM training) and learning without concurrent tDCS (sham).

Effects of the stimulation on the follow-up session of the training task and the transfer effects (on T4 and follow-up) were examined by submitting the performance outcome (mean n and d’) of the training- and 3-backs task to ANCOVAs with the performance at the respective training session (T4 or follow-up) as dependent variable, baseline performance as covariate and group as between-subjects factor^83^. When significant effects were revealed, regression parameters of the underlying linear regression were reported to identify the specific impact of the significant variables.

Correlations between training task baseline performance of participants and the sample demographic data were evaluated by a simple linear regression with baseline performance as dependent variable and age, sex, education, right-handedness and IQ as predictors. Blinding of group allocation was checked via a χ^2^-Test. Changes of affect as assessed by the PANAS were analyzed for the positive and negative scale separately using an ANOVA on change scores with training session (T1-T3) as within-subjects variable, group as between-subjects variable and the session × group interaction. Kruskal-Wallis tests for each aspect of adverse effect were calculated with group as between-subject factor to control for differences between groups. Regarding the effect size of main effects and interactions of the ANCOVAs, Eta-squared (η^2^) and partial Eta-squared (η_p_^2^) are reported^84^. Cohens d was calculated for all post-hoc t-Test comparisons. For all analyses the alpha level of statistical significance was set to p < 0.05.
